# Study of the Effects on the Strengthening Mechanism and Wear Behavior of Wear-Resistant Steel of Temperature Controlling in Heat Treatment

**DOI:** 10.3390/nano14141171

**Published:** 2024-07-09

**Authors:** Xiaoyu Zhu, Jianghai Lin, Shaoning Jiang, Aijun Cao, Yuan Yao, Yu Sun, Sensen Li, Zhanfeng Zhang

**Affiliations:** 1 School of Mechanical Engineering, Qilu University of Technology, Shandong Academy of Sciences, Jinan 250031, China; zxy786040500@163.com (X.Z.); ljh6099@163.com (J.L.); 2 Shandong Institute of Mechanical Design and Research, Jinan 250353, China; 3 Shantui Construction Machinery Co., Ltd., Jining 272073, China; 4 HIMILE Mechanical Science and Technology Co., Ltd., Weifang 261500, China; wy13682024@126.com (A.C.); fzghb@himile.com (Y.Y.); 5 Shandong Sun Wearparts Co., Ltd., Jining 272000, China; lexianju0851epo@126.com (Y.S.); jpg8813012589913@163.com (S.L.); 6 Shandong Jinhengli Machinery Manufacturing Co., Ltd., Taian 271208, China; oinmte5148@outlook.com

**Keywords:** wear-resistant steel, temperature controlling, microstructure, mechanical property, wear resistance

## Abstract

To improve the wear resistance of the materials used for blades in engineering machinery, this study focused on the microstructural characteristics, mechanical properties, and wear behavior of HB500 grade wear-resistant steel developed using an optimized heat treatment system. To improve the temperature uniformity of the heat treatment furnace, the method of cyclic heating was used to heat the components. Carefully designing the quenching equipment, such as using a cross-shaped press, was employed to enhance the quenching effect and reduce the deformation of the steel plates. The crystal orientation analysis revealed a uniform and fine-grained microstructure, primarily characterized by plate-type tempered martensite, which indicated a good hardenability. The microstructure observations showed that the width of martensite is approximately 200 nm, with a significant presence of dislocations and carbides. Tensile tests and multi-temperature gradient impact tests indicated superior mechanical properties compared to similar grade wear-resistant steels, including a Rockwell hardness of 53, tensile strength of 1610 MPa, yield strength of 1404 MPa, and total elongation around 12.7%. The results of friction and wear experiments indicate that the wear rate decreases as the load increases from 100 N to 300 N, demonstrating an excellent wear resistance under a large load. Observations of the worn surfaces indicated that the wear mainly involved adhesive wear, fatigue wear, and oxidative wear. The properties’ improvements were attributed to microstructure refinement and precipitation strengthening. This study indicates that designing a heat treatment system to control temperature uniformity and stability is feasible.

## 1. Introduction

Martensitic wear-resistant steel is commonly used for engineering machinery such as cutting edge plates due to its excellent wear resistance [1,2]. Currently, martensitic wear-resistant steels mainly include Hardox from Sweden’s SSAB (Oxelösund, Sweden), XAR from Germany’s ThyssenKrupp (Neuss, Germany), and Raex from Finland’s Ruukki (Helsinki, Finland) and the NM series in China [3,4,5]. As key components in bulldozers and similar equipment’s, serious wear remains a crucial problem to limit the service life of cutting edge plates. Therefore, further improving the properties of martensitic wear-resistant steel is an urgent need.

The improvement of these properties should start with the refinement of the microstructure or formation of precipitates. Adjusting the elemental composition of materials to improve the properties of martensitic wear-resistant steel is a commonly used method. For example, the addition of Mn can improve hardenability through the refinement of ferrite and pearlite [6]. The addition of Cr promotes the generation of nano-sized precipitates, leading to grain refinement and thereby enhancing the material’s strength [7]. The addition of Al can refine grains and improve wear resistance while deoxidizing [8]. Cu, in martensitic aging steels, promotes the formation of nano-sized precipitates, improving the material’s mechanical properties through lattice mismatch between precipitates and the matrix [9]. Although adding alloying elements is an effective strategy, most of them will increase the production costs of factories, and their additions are limited to within certain ranges.

The optimization of heat treatment process is another key method for improving the comprehensive properties of materials. In order to improve wear resistance, quenching and tempering are carried out to obtain a martensitic microstructure which provides a high hardness to wear-resistant steel. Research has found that the optimization of the parameters during the quenching and tempering processes, including but not limited to an increased quenching temperature [10], a tempering temperature exceeding 300 °C [11], and an increased cooling rate, can increase hardness, tensile strength, toughness, and then wear resistance [12]. The improvement of such properties through parameter optimization during heat treatment as well as a composition adjustment is related to the evolution of the microstructure through the refinement of grain or martensite and the precipitation of carbides [11,13,14]. Han effectively enhanced the mechanical properties and wear resistance of martensitic steel by controlling grain elongation and increasing the austenite dislocations density through low-temperature hot rolling [15]. Tang adjusted the density of κ-carbid through an aging treatment, increasing the strength and hardness of FeMnAlC steel, resulting in a good wear resistance under low loads [16]. Thus, it can be seen that microstructural refinement and precipitation play important parts in improving the properties of wear-resistant steels.

It should be pointed out that most research focuses on controlling material properties in the laboratory. Under the same heat treatment parameters, the material properties obtained from laboratory and large-scale production often show certain discrepancies, mainly in performance stability. In large-scale factory production, controlling costs is the most important. And it is often difficult to control heat treatment parameters precisely, leading to coarse microstructures and subsequent unstable properties in products.

A stable and innovative heating and cooling system to control temperature during heat treatment is necessary to obtain the desired microstructure under the precondition of reducing production costs. Based on the above analysis, wear-resistant materials for cutting edge plates were researched by cooperating with a related company to design a heating and cooling system. Cyclic heating ensures a more uniform temperature rise during the heating process, while an induction quenching process and cross-shaped press technology allow for more thorough quenching. The microstructure characterization, the mechanical properties, and the wear behavior were investigated. And the desired stable and comprehensive properties were obtained without increasing the cost.

## 2. Experimental Materials and Methods

### 2.1. Material Preparation Process

The experimental material is HB500 wear-resistant steel (HB500 in short, Shandong Sun Wearparts Co., Ltd., Jining, China), one grade of commercial martensitic wear-resistant steel classified according to Brinell hardness [17,18]. The designed and measured compositions are shown in Table 1. Converter smelting with a nominal capacity of not less than 120,000 kg was used to smelt the steel. The off-furnace refining we adopted was Ladle Furnace (LF−120t, Chengde Jianlong Special Steel Co., Ltd., Chengde, China) + Ruhrstahl Heraeus Degassing (RH−120t, Chengde Jianlong Special Steel Co., Ltd., Chengde, China) vacuum degassing refining. The specific parameters of the rolling process are as follows: heating temperature was 1200 °C, final rolling temperature was 950 °C, cooling speed was controlled at 15 °C/s, and initial steel plates with a thickness of 20 mm were obtained after final rolling.

**Figure 1:**
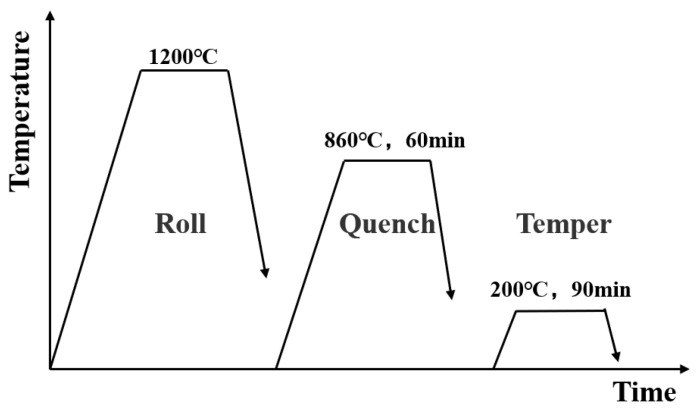
Schematic diagram of HB500 wear-resistant steel rolling and heat treatment process.

After punching to the shape of the cutting edge, the steel plates underwent quenching (860 °C/60 min, water cooling) and tempering (200 °C/90 min, air cooling) heat treatments [19]. A schematic diagram of the rolling and heat treatment processes is shown in Figure 1.

**Figure 2:**
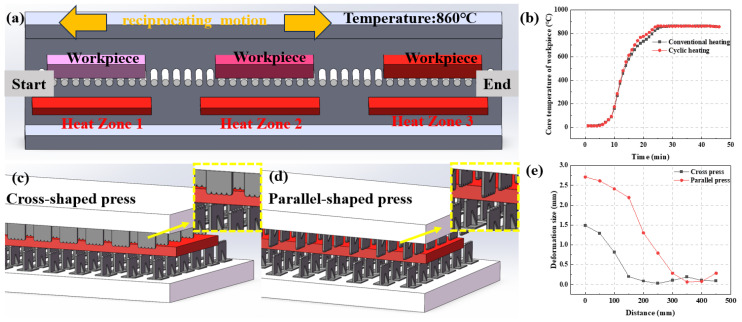
Schematic diagram of cyclic heating process (**a**) and induction quenching (**c**,**d**), and the related comparative data of core temperature (**b**) and deformation (**e**).

During heat treatment, a cyclic heating process was designed, as shown in Figure 2a, in which steel plates underwent reciprocating motion in different heating zones of the furnace. During heating, the steel plate moves sequentially through the furnace, which can result in uneven heating or overheating. In the improved furnace, different temperature zones were created. The steel plate moves to the next zone only after reaching the set temperature in the current zone. If a steel plate does not meet the temperature criteria, it is sent back to the previous zone to repeat the process. In comparison to conventional sequential heating, cyclic heating can enhance the overall heating rate of the material and improve the uniformity of core temperature, as depicted in Figure 2b.

When the steel plate reached the set temperature, a cooling system including the induction quenching process and a cross-shaped press structure was incorporated as shown in Figure 2c,d. The induction quenching section adjusted the quenching process parameters accurately based on the sensor-detected thickness of the steel plate. It precisely controls the quenching water consumption, flow rate, pressure, and spray time, enhancing quenching efficiency while conserving water. The quenching parameters are shown in Table 2.

The steel plate was quenched using a pressurized method. During quenching, the plate is clamped between upper and lower pressing plates, shown as gray components in Figure 2c,d, with the red part representing the steel plate. The quenching liquid is sprayed onto the plate surface from between the pressing plates. Traditional parallel press beds have upper and lower pressing plates arranged in parallel, with a larger gap between them. In our study, the pressing plates are arranged vertically, increasing the contact area with the steel plate and reducing deformation during cooling. A comparison of deformation between the two presses under the same quenching parameters is shown in Figure 2e; the deformation of the steel plate using a traditional parallel-shaped press is larger, at 2.7 mm, and after using the cross-shaped press, the deformation of the steel plate is reduced to 1.5 mm. Therefore, using a cross-shaped press to press the steel plate results in better hardening effects.

After the heat treatment, microstructure observation and properties tests were carried out on the cutting edge plates, including crystal orientation analysis busingy Electron Backscatter Diffraction (EBSD), microstructure observation using a Transmission Electron Microscope (TEM), and properties analysis through hardness test, tensile tests, Charpy V-notch impact tests, and friction and wear tests. The details are as follows.

### 2.2. EBSD Observation

Thin slices with 10 mm × 10 mm × 2 mm were cut using an electric spark line cutting machine. The samples were mechanically polished with 600#, 1000#, 2000#, and 3000# SiC sandpaper in sequence. Subsequently, electro-polishing was performed at room temperature (RT) to remove the surface deformation layer caused by mechanical grinding, using a polishing solution composed of 7% high chlorate acid and 93% ethanol, with a current density of 450 mA/cm^2^, for approximately 1 min. EBSD observations were performed using EDAX Velocity Super (EDAX Inc., Mahwah, NJ, USA). The texture and orientation analyses of the samples were conducted using Energy Dispersive X-ray Spectroscopy (EDAX) Velocity Super. The central region of the samples was chosen for testing, with a step size of 0.1 μm and an actual testing area of 50 × 50 μm.

### 2.3. TEM Observation

TEM samples were thin disks with a diameter of φ3 mm, similarly cut using an electric spark line cutting machine and ground to a thickness of 0.1 mm using sandpaper. Then, the samples were prepared using twin-jet electropolishing device, with an electrolyte solution consisting of 5% high chlorate acid and 95% ethanol and an electrolysis voltage of 25–28 V, at −20 °C. The samples were observed using FEI Talos F200X type TEM (Talos Technology Co., Ltd., Shenzhen, China), and Digital Micrograph software (DM 3.2) was used for data analysis.

### 2.4. Mechanical Property Test

Hardness tests were conducted using a Rockwell hardness tester (Taiming Optical Instrument Co., Ltd. of Shanghai, Shanghai, China). Tensile tests were conducted according to GB/T228.1-2021 on standard tensile specimens with a gauge length of 50 mm and a diameter of φ5 mm, using a WDW-300E (Suzhou Nanguang Electronic Technology Co., Ltd., Suzhou, China) universal testing machine at a constant speed of 0.5 mm/min at RT [20]. The model of the extensometer is YYU-10/50 (The NCS Testing Technology Co., Ltd., Beijing, China). Charpy V-notch impact tests were performed on standard specimens (10 mm × 10 mm × 55 mm) processed according to GB/T229-2020 at RT to −50 °C using a ZBC2452-C (Meters Industrial Systems (China) Co., Ltd., Shenzhen, China) [21]. The fracture morphology of the samples was observed using a JEOL JSM 7200F type Scanning Electron Microscope (SEM) (JEOL Ltd., Tokyo, Japan).

### 2.5. Wear Resistance Test

**Figure 3:**
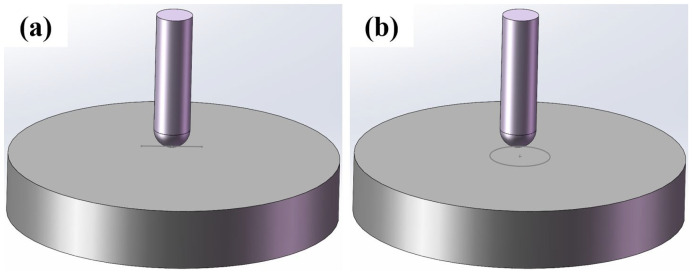
Schematic diagram of friction and wear test under different modes: (**a**) reciprocating mode and (**b**) rotating mode.

Wear resistance tests were carried out using a UMT-3 (Brookfield Engineering Laboratories, Inc., Waltham, MA, USA). The abrasive materials were φ6 mm GCr15 steel balls and φ55 mm × 10 mm HB500 steel disks. The sample surfaces were polished smooth. Loading forces of 100 N, 200 N, and 300 N were applied for 20 min, and the tests were conducted in reciprocating and rotating modes. In reciprocating mode, the travel distance was 10 mm with a frequency of 1 Hz, while in the rotating mode, the rotation diameter was 10 mm with a speed of 200 rpm. A schematic diagram of the friction and wear test is shown in Figure 3, where the gray line represents the wear track. After the tests, the samples were analyzed for wear morphology and wear volume using a Bruker Contour GT-K (Brookfield Engineering Laboratories, Inc., Waltham, MA, USA).

## 3. Results and Discussion

### 3.1. EBSD Analysis

**Figure 4:**
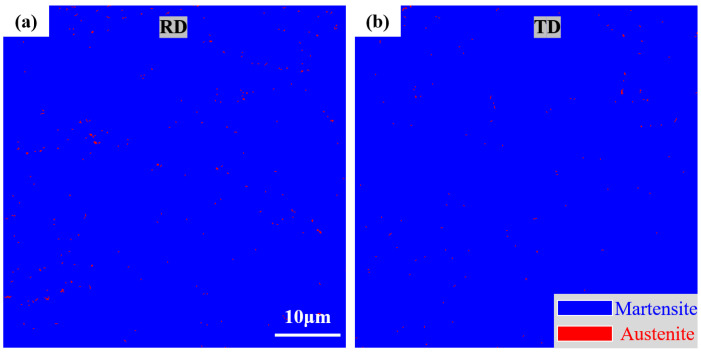
Phases constituting the HB500 in (**a**) RD and (**b**) TD.

To analyze the structural and orientation characteristics of optimized HB500, EBSD observations were conducted on samples in two directions, the rolling direction (RD) and transverse direction (TD), providing a detailed characterization of their structure and grain boundaries [22]. Figure 4 illustrates the phases constituting the two directions. The blue regions represent martensite structure, with a minimal amount of red indicating residual austenite. The statistical analysis revealed a residual austenite content of 0.278% in the RD and 0.146% in the TD, indicating a uniform structure and good hardenability for HB500.

**Figure 5:**
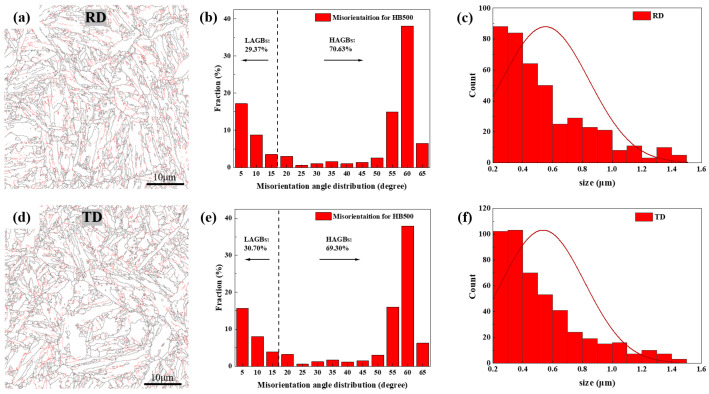
Statistics for grain boundaries, orientation differences, and grain size of HB500 in two directions: RD (**a**) grain boundaries, (**b**) distribution of orientation differences, and (**c**) grain size; TD (**d**) grain boundaries, (**e**) orientation difference distribution, (**f**) grain size.

Figure 5 displays statistics on grain boundaries and orientation differences in the two directions for HB500. High-angle grain boundaries (greater than 15°, HAGB in short) are marked in black, while low-angle grain boundaries (usually dislocations, less than 15°, LAGB in short) are marked in red. As shown in Figure 5a,d, clear grain boundaries and martensite structures are presented, forming elongated plate-like martensite. The statistical analysis (Figure 5b,e) reveals that large-angle grain boundaries account for approximately 70%. The grain size statistics (Figure 5c,f) show an average grain size of 0.55 μm in the RD and 0.53 μm in the TD, with most sizes being below 0.4 μm. The grain sizes in the RD and TD exhibit minimal differences, indicating a uniform structure.

**Figure 6:**
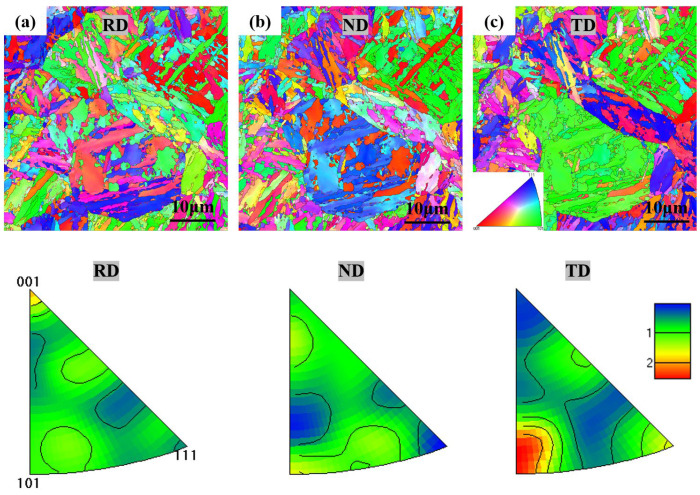
IPF map of HB500 in different directions (**a**) RD, (**b**) ND and (**c**) TD.

Further orientation analysis reveals a consistent orientation in various directions for HB500. Figure 6 shows the inverse pole figure (IPF) orientation map of the sample, indicating consistent crystal orientations within grains, contributing to the material’s stable microstructure [23]. As depicted in Figure 6, the orientation intensity in the RD and Normal Direction (ND) is uniform, with a weak texture in TD along (101) but with a low intensity and no obvious preferred orientation.

### 3.2. TEM Analysis

**Figure 7:**
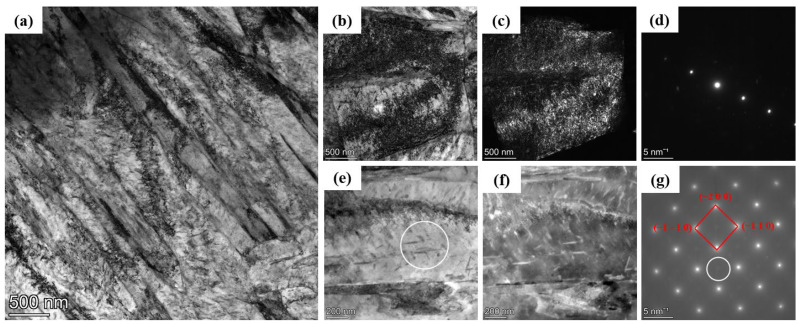
Microstructure of HB500: (**a**) morphology of HB500; (**b**) BF image, (**c**) DF image, and (**d**) double-beam diffraction in dislocations; (**e**) BF image, (**f**) DF image, and (**g**) SAED in precipitates.

The EBSD results show that the HB500 has a uniform structure. Further characterization was performed using TEM, as shown in Figure 7. In Figure 7a, the presence of plate-like martensite and dislocations is clearly observed [24], with a martensite plate width of around 200 nm. Additionally, as shown in Figure 7b–d, the dislocations within the plates are characterized using bright-field (BF), dark-field (DF) and Selected Area Electron Diffraction (SAED) modes, revealing bent and entangled high-density dislocations. Crystal defects like dislocations can nucleate more martensite, thereby refining the martensite plates [25]. The movement of dislocations within the plates would affect the deformation behavior of martensitic steel and result in improved strength and excellent mechanical properties [26]. The results for the steel’s mechanical properties are discussed in Section 3.3.

**Figure 8:**
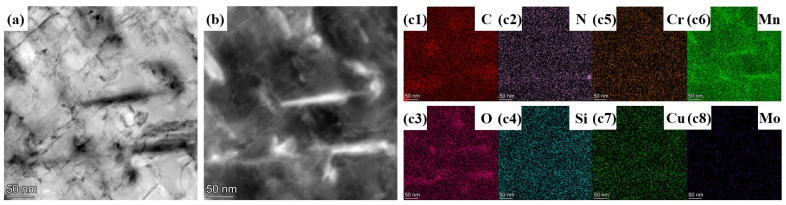
Precipitate phase in HB500: (**a**) BF image, (**b**) DF image, element distribution (**c1**) C, (**c2**) N, (**c3**) O, (**c4**) Si, (**c5**) Cr, (**c6**) Mn, (**c7**) Cu, (**c8**) Mo.

In addition to dislocations, the presence of a precipitation phase is observed with a width of 10–20 nm and an area fraction of 2.59%, which is confirmed by the BF image, DF image, and corresponding SAED shown in Figure 7e,f. To further confirm the elemental distribution of the precipitation phase, element mapping was performed as shown in Figure 8. Combined SAED analysis, the elemental distribution indicates that the main component in the precipitation phase in HB500 is likely to be Mn_23_C_6_.

**Figure 9:**
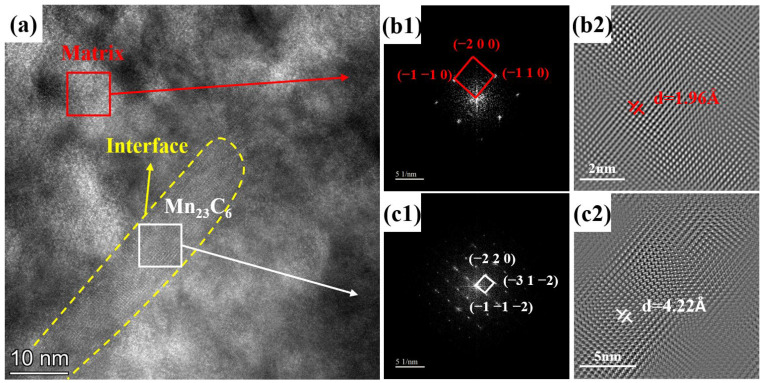
HRTEM image of HB500 (**a**), (**b1**) FFT and (**b2**) IFFT images of matrix, (**c1**) FFT and (**c2**) IFFT images of precipitate phase.

By utilizing high-resolution transmission electron microscopy (HRTEM) images for Fourier transform (FFT) followed by inverse Fourier transform (IFFT), a more precise lattice image was obtained, as illustrated in Figure 9. The FFT results indicate that both the matrix and precipitates possess a body-centered cubic (BCC) crystal structure. The measured *d* values (lattice parameter) for the matrix and precipitates in IFFT were 1.96 Å and 4.22 Å, respectively.

### 3.3. Mechanical Property Analysis

After designing the heating and cooling system, fine grains, tempered martensite with narrow plates, precipitates, and high-density dislocation were obtained. Based on the microstructural characterization results, mechanical property analysis of HB500 was conducted. The average Rockwell hardness (HRC) for HB500 is 53 (Table 3).

**Figure 10:**
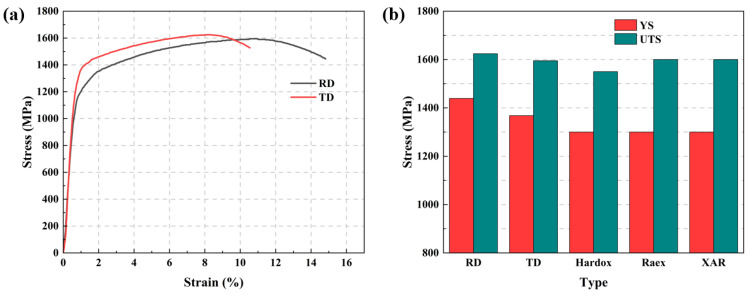
(**a**) Stress–strain curve of HB500 and (**b**) comparison of tensile performance [29,30,31].

Tensile tests were performed on the selected wear-resistant steel in both the RD and TD. A stress–strain curve is obtained by processing the deformation data of the extensometer and the force data of the tensile machine, as shown in Figure 10a. It is observed that the yield strength and tensile strength are similar in both directions, with the elongation in the RD being greater than that in the TD, aligning with the characteristics of steel post-rolling.

Figure 10b compares the stress of HB500 in this study with those existing commercial HB500 grade wear-resistant steel, Hardox, Raex, and XAR [27]. The tensile strengths are roughly the same, but the yield strength in this study is superior, which may be related to the refinement of grain and martensite and the formation of precipitates discussed below [28].

**Figure 11:**
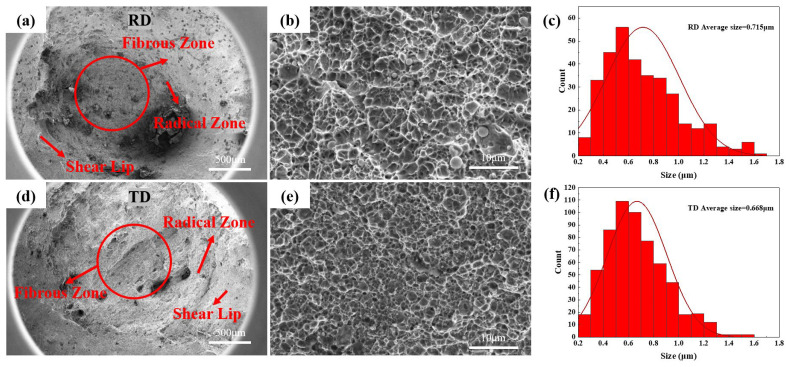
SEM morphology and dimple width statistical macroscopic morphology of tensile specimens in (**a**) RD and (**d**) TD; partial enlargement in (**b**) RD and (**e**) TD; and statistics for dimple width in (**c**) RD and (**f**) TD.

The fractured surfaces of the tensile samples were characterized, as shown in Figure 11. The macroscopic morphology, as illustrated in Figure 11a,d, reveals an obvious fibrous zone, a less obvious radical zone [32], and dimples in the fibrous zone [33]. Fracture analysis shows numerous dimples and some depressions, without cleavage facets, indicating the presence of a ductile fracture [34]. Dimensional statistics of the dimples, as seen in Figure 11c,f, show the average width of dimples to be 0.715 μm in the RD and 0.668 μm in the TD. Larger dimples correlate with increased elongation [35].

**Figure 12:**
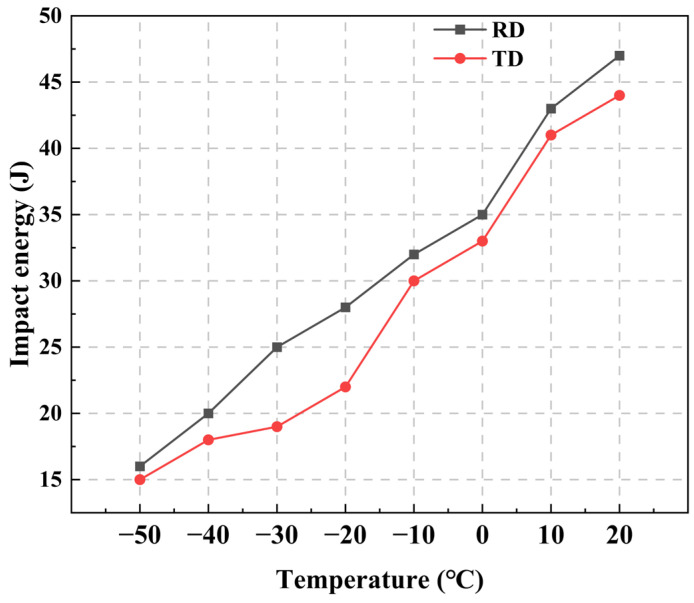
Impact energy curves of HB500 in RD and TD.

**Figure 13:**
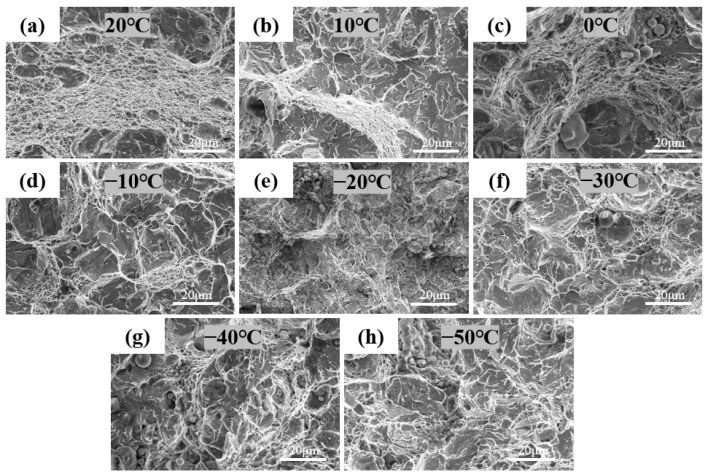
The fracture morphology of HB500 impact specimens at different temperatures: (**a**) 20 °C, (**b**) 10 °C, (**c**) 0 °C, (**d**) −10 °C, (**e**) −20 °C, (**f**) −30 °C, (**g**) −40 °C, (**h**) −50 °C.

Impact tests at different temperatures were conducted on samples in the RD and TD, and the corresponding impact energies are shown in Figure 12. It can be observed that the impact performance in RD and TD is similar and the impact energy of the material at room temperature is approximately 45 J, and has an impact energy of 15 J until −50 °C. The Hardox 500 steel energy absorbed at −40 °C was 37 J. In this study, HB500 still has a gap in its low-temperature impact toughness compared to Hardox Steel. Fracture analysis was performed on impact samples in the RD, and SEM morphology observations of fracture surfaces at different temperatures are shown in Figure 13.

From Figure 13a–d, it can be seen that the fracture morphology is primarily characterized by dimples mixed with a small number of cleavage planes. As the temperature decreases, the proportion of dimples also reduces.

The improvement in mechanical properties is mainly because HB500 has achieved the refinement of the grains and martensite and the formation of precipitates by controlling temperature uniformity and stability. The generation of large-angle grain boundaries and a smaller grain size in martensitic steel can effectively hinder crack propagation [14,16]. This is attributed to the smaller grain size required to traverse more large-angle grain boundaries when cracks occur, increasing energy consumption and improving strength and toughness [36,37,38]. The strengthening mechanism caused by the fine grains can be described by the Hall–Petch equation [39,40,41]. The formation of precipitates increases the material’s dislocation density, while more dislocations can nucleate more martensite, thereby refining the martensite plates [25]. In the interaction between dislocation and refined martensite plates, the extension of material during fracture is hindered, leading to an increase in hardness and strength [42,43,44]. Meanwhile, the presence of nano-sized precipitates impeded the dislocation motion and alleviated the localized stress concentration in the dislocation slip, thereby delaying crack initiation [45,46,47] and enhancing the material’s strength [48,49].

### 3.4. Wear Resistance Analysis

**Figure 14:**
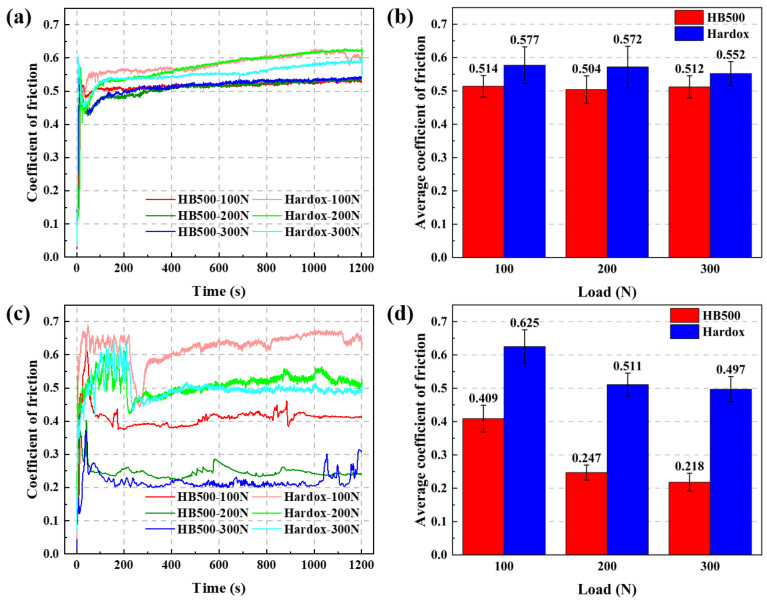
Friction coefficient curves and average friction coefficient of HB500 and Hardox under different loads: (**a**,**b**) reciprocating mode; (**c**,**d**) rotation mode.

To visually evaluate the wear resistance of HB500, reciprocating and rotating experiments were conducted on HB500 and Hardox. The friction coefficient, wear volume, and wear rate were studied. Figure 14 shows the friction coefficients and average friction coefficients of HB500 and Hardox under two modes. From Figure 14a,c, it can be seen that the friction coefficient varies greatly with time, but overall, it shows a regular pattern. In the initial stage of friction, the contact area between the rubbing materials is small and the materials are not fully in contact, resulting in a lower friction coefficient [50,51]. After 200 s of wear, i.e., the end of the running-in period, the contact area stabilizes and enters the stable friction stage, and the measured friction coefficient also stabilizes [52]. The wear debris generated during the wear process is oxidized and hardened into high-hardness particles, which are mixed in the contact area, resulting in small fluctuations in the friction coefficient. From Figure 14b,d, it can be seen that the friction coefficient in reciprocating mode is not significantly affected by the load, and the friction coefficients are roughly equal under three different loads. However, in the rotating mode, the friction coefficient decreases with the increase in the load. Compared with Hardox, it can be found that the friction coefficient of HB500 is lower than that of Hardox, with a small difference in the reciprocating mode, but a difference of about 50% in the rotating mode. In the reciprocating mode, the movement between the contact surfaces is back-and-forth, causing the stress on the material surface to constantly change in two directions. In contrast, in the rotating mode, the movement of the contact surfaces is continuous and unidirectional, resulting in a sustained, unidirectional frictional force on the material’s surface. This frictional behavior may amplify the differences in the inherent wear resistance of the materials. Consequently, there will be significant differences in the friction coefficients under different friction modes.

**Figure 15:**
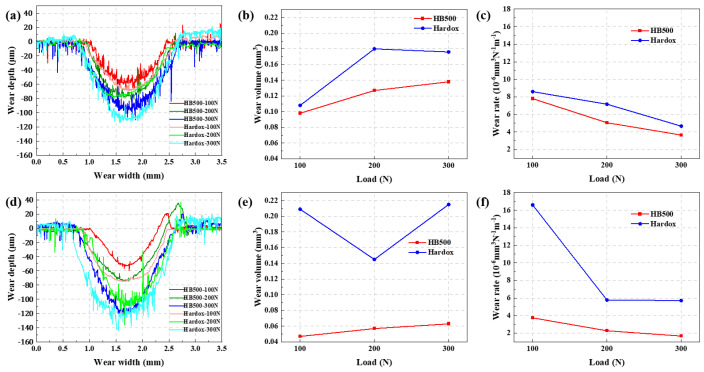
White light experimental data under different loads in reciprocating mode: (**a**) wear profile, (**b**) wear volume, and (**c**) wear rate; in rotation mode: (**d**) wear profile, (**e**) wear volume, (**f**) wear rate.

In addition to comparing the friction coefficients, a white light observation was performed on the surfaces of HB500 samples. Figure 15a,d display the contour data exported from the white light experiments, illustrating the wear track contour data under different loads and experimental modes. It is observed that there are protrusions along the edges of the wear tracks, which are most pronounced in the rotating mode and can attributed to the pronounced accumulation caused by the centrifugal force during the rotation of the abrasive ball [53]. It is evident that the wear depth increases with the increase in load. In the reciprocating mode, the degree of wear growth decreases with increasing load, suggesting that the impact of material wear surface under high loads gradually diminishes, providing preliminary evidence of good wear resistance. Moreover, in both friction modes, the wear depth of HB500 was consistently lower than that of Hardox, indicating a superior wear resistance.

To quantify the wear behavior more intuitively, the wear volumes were statistically compared, as shown in Figure 15b,e. The Archard formula [54] was used to calculate the wear rates under two modes, as illustrated in Figure 15c,f:

(1) W=V/NL

where *W* is the wear rate, *V* is the wear volume, *N* is the load, and *L* is the total wear distance.

As can be seen from Figure 15c,f, with increasing load, the wear rate decreases in both modes to become insignificant in HB500 and Hardox. In comparison with Hardox, it is found that the wear rate of HB500 is lower. This indicates that HB500 exhibits good wear resistance in both friction modes.

**Figure 16:**
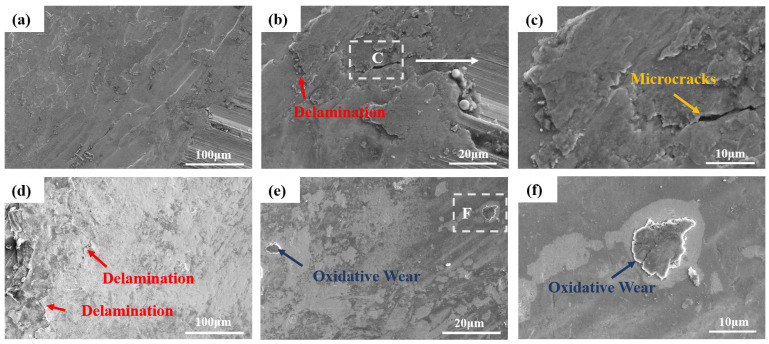
SEM morphology of friction and wear of HB500 under two modes. Reciprocating mode: (**a**) low magnification, (**b**) high magnification, (**c**) local amplification; rotation mode: (**d**) low magnification, (**e**) high magnification, (**f**) local magnification.

To further analyze the wear mechanism, a comparison of the worn surfaces of the HB500 samples under a 100 N load in both modes was conducted to observe their morphologies, as depicted in Figure 16. From Figure 16a,d, it can be seen that most of the wear bottoms exhibit relatively flat surfaces due to the high hardness of HB500. Simultaneously, due to the abrasive action during the experiment, the abrasive material exerted pressure on the substrate during friction, resulting in partial plastic deformation at the interface between the wear track and the substrate. These deformed areas, under high loads, promote the generation of microcracks, ultimately leading to delamination [51], which is indicated by red and yellow arrows in Figure 16. Repeated cyclic shearing forces in these regions easily induce stress concentration, pitting, and crack formation. As the pits and cracks expand, this specific area peels off from the material substrate, resulting in adhesive and fatigue wear. In addition, as shown in Figure 16e, a small amount of oxidized wear flaking areas is observed on the worn surface in the rotating mode. This can be attributed to the layering caused by the growth of an oxide film during the wear test reaching a critical thickness and subsequently delaminating under the influence of frictional forces [55], indicated by blue arrows.

Through the above analysis, comprehensive properties were obtained for HB500 by temperature controlling during the heating and cooling process. The improvement in the properties can be seen from the high hardness, strength, and good wear resistance. A high hardness enhances the material’s ability to resist wear, reducing the damage to the worn surface and the generation of abrasive particles during the wear process, thereby improving the material’s wear resistance [56,57]. A high strength indicates that materials have high resistance to deformation, which can make the matrix resist the invasion of abrasive particles during wear and reduce the occurrence of plowing during the wear process [58]. Due to the improvement of the above properties, HB500 is difficult to intensify wear due to external forces and abrasive particles during the wear process, thereby reducing the detachment of abrasive particles and improving overall wear resistance.

However, almost all of the tests were carried out at room temperature, which does not give an idea of how the wear resistance changes in low temperatures. Based on this investigation, potential future research could be conducted to determine how well the material performs in low-temperature settings.

## 4. Conclusions

The strengthening mechanism and wear behavior of a HB500 grade wear-resistant steel was studied by temperature controlling during the heating and cooling process. The influence of fine grain and martensite strengthening and precipitation strengthening on its properties has been analyzed. Our conclusions are summarized as follows:

After heat treatment, a uniform structure was obtained which consisted of narrow-plate martensite with fine grains, high-density dislocations, and precipitates. The average grain size was 0.55 μ m, the width of the precipitated phase was 10–20 nm, and the fraction area was 2.59%. The overall mechanical properties of the steel were obtained, including a high hardness and strength, as indicated by a Rockwell hardness of 53, tensile strength of 1610 MPa, yield strength of 1404 MPa, and total elongation around 12.7%. The improvement in the steel’s properties can be seen from its good wear resistance. The wear resistance was improved according to the friction coefficient, wear volume, and wear rate.

The improvement in the properties of HB500 wear-resistant steel is attributed to the refinement of fine grain and martensite and the formation of precipitates. The HB500 wear-resistant steel we have researched has been used for cutting edge plates. It not only has good mechanical properties, but also an improved wear resistance, thus greatly improving its service life and work efficiency.

## Figures and Tables

**Table 1:** Chemical composition of the HB500 (wt.%).

	C	Si	Mn	P	S	Cr	Cu	B	Ti	Al	Fe
Design	0.26–0.31	0.15–0.35	1.1–1.45	<0.02	<0.01	0.3–0.6	<0.3	0.0005–0.003	0.018–0.05	0.015–0.04	Bal.
Reality	0.27	0.21	1.19	0.017	0.007	0.37	0.017	0.001	0.03	0.038	Bal.

**Table 2:** The quenching parameters of wear-resistant steel plates.

Thickness (mm)	12	20	25	30	50	60
Water consumption (L)	5320	8866	11,083	13,299	22,166	26,969
Spray time (s)	64	106	132	160	266	320

**Table 3:** The Rockwell hardnesses of HB500.

Sample	1	2	3	4	5	Average
Value	53.4	52.6	54.8	52.6	51.7	53.02

## Data Availability

The datasets analyzed during the current study are not publicly available due to the nature of this research, but are available from the corresponding author on reasonable request.
